# Developing an Aptamer-Based Colorimetric Method for Highly Specific Discrimination of Hg^2+^ and MeHg

**DOI:** 10.3390/molecules31213925

**Published:** 2026-11-01

**Authors:** Zhiqiang Chen, Kangyao Zhang

**Affiliations:** 1 School of Food Engineering, Zhangzhou Institute of Technology, Zhangzhou 363000, China; 2 School of Advanced Manufacturing, Fuzhou University, Quanzhou 362251, China; zhangky@fzu.edu.cn

**Keywords:** food safety, mercury pollution, aptamer, gold nanorods, colorimetric method

## Abstract

Highly toxic inorganic mercury (Hg^2+^) and methylmercury (MeHg) are widespread heavy metal pollutants that negatively affect human health and ecosystems. Thus, developing a method for the rapid, facile, and visual detection of Hg^2+^ and MeHg to prevent mercury poisoning is urgent and challenging. In this study, a colorimetric biosensor using a thymine-rich (T-rich) H_T9_ deoxyribonucleic acid (DNA) aptamer serving as a recognition probe (RP) labeled with a thiol group (-SH) and poly diallyldimethyl ammonium chloride (PDDAC)-coated gold nanorods (AuNRs) as a signal probe (SP) was developed for the specific detection of Hg^2+^ and MeHg. In the presence of Hg^2+^, Ag^0^/Hg^0^ amalgam nanoparticles (ANPs) were formed and bound to the H_T9_ DNA aptamer, producing a light lavender color. Due to its stronger binding ability, MeHg showed priority binding to the H_T9_ DNA aptamer, yet reduced Ag^0^ NPs were deposited on the surface of PDDAC-coated AuNRs. Interestingly, this produced a color ranging from olive to atrovirens. The method exhibited a good linear relationship in ranges of 0.30–1.00 μM and 0.50–5.00 μM for Hg^2+^ and MeHg, respectively, with corresponding visual limits of detection (LODs) of 0.30 μM (for Hg^2+^) and 0.50 μM (for MeHg). Their theoretical LODs (3σ/S) were calculated to be 0.071 μM (for Hg^2+^) and 0.12 μM (for MeHg). The method used in this work was successfully applied to the colorimetric detection of Hg^2+^ and MeHg in real seawater samples, with a recovery (Rec.) of 98–107% and a relative standard deviation (RSD, n = 6) ≤ 8%. This method provides a useful technical reference for the semi-quantitative determination of Hg^2+^ and MeHg in drinking water and aquatic products, especially in the case of accident-level contamination.

## 1. Introduction

Mercury is a known pollutant worldwide; ubiquitous and highly toxic, it poses a significant threat to the environment, food, and humanity. Currently, there is a serious situation concerning global mercury pollution, as 6000 to 8000 t of mercury is emitted into the atmosphere yearly, and the environmental mercury deposition induced by anthropogenic emissions has recently increased by up to five times [1,2]. Such large-scale mercury emissions result in the widespread distribution of mercury in the environment, such as in the air, soil, and water. Meanwhile, microorganisms convert inorganic mercury (Hg^2+^) into more toxic methylmercury (MeHg) or ethylmercury (EtHg), resulting in increasingly higher bioaccumulation through the food chain and, eventually, severe biomagnification occurring in apex predators like humans [3,4,5]. The stability of the ecosystem structure is hence disrupted, resulting in embryotoxicity and immunodeficiency in animals, and neurotoxicity and metabolic disorders in aquatic organisms; in addition, strong neurotoxicity and irreversible developmental damage to susceptible populations can also result from the ingestion of mercury-contaminated crops and seafood [6,7]. Due to its extreme toxicity, robust persistence, and the potential risks from food chain transfer, precise determination is particularly important to prevent mercury poisoning and monitor mercury levels in the environment, food, and the human body. Countries worldwide have put many restrictive measures in place regarding the use of mercury-containing products [8,9,10,11,12]. Thus, developing rapid, reliable, and low-cost detection technologies plays a pivotal role in ensuring food safety and protecting human health and ecological stability.

Real samples that have undergone the necessary pretreatment can be analyzed using various technologies. However, early, conventional methods of detecting mercury rely primarily on single or hyphenated instrumental analysis, such as atomic absorption spectrometry (AAS) and high-performance liquid chromatography–inductively coupled plasma–mass spectrometry (HPLC-ICP-MS), approaches that possess high sensitivity and are capable of simultaneous multi-element detection. These instruments, however, have their drawbacks: they are expensive and complex, require a professional to operate them, and their analysis is time-consuming and results in a high cost [13,14]. Most crucially, they are difficult to use to directly distinguish MeHg (or organic mercury) from total mercury content, leaving them unable to meet the needs for on-site rapid detection and specific analysis of mercury species [15,16,17]. To overcome the above limitations, researchers have developed colorimetric, electrochemical, fluorescent, chemiluminescent, and surface-enhanced Raman spectroscopy (SERS), among other emerging methods [18]. Amongst these techniques, colorimetric methods are highly favored because of their easy observation, low cost, and simple operation. Based on the specific interaction between chromogenic reagent and mercury ions, color-rendering and absorption spectral change are triggered by forming nanoparticle aggregations, metal amalgams, or thymine–Hg^2+^–thymine (T-Hg^2+^-T) coordination [19,20,21,22]. However, the performance of traditional colorimetric methods in terms of their selectivity, sensitivity, detection limit, and interference immunity still requires improvement.

Gold nanorods (AuNRs) are a class of colloidal nanomaterials with a unique anisotropic shape, high specific surface area, tunable plasmonic properties, and nonpositive Gaussian curvature [23,24,25]. The simple surface chemical properties are conducive to multiple functional modifications on their surface [26]. Compared with other gold nanomaterials, AuNRs exhibit excellent stability, optical sensitivity, and application potential in disease treatment, drug delivery, and bioimaging [27,28]. In recent years, numerous mycotoxins, small-molecule metabolites, pesticide residues, and veterinary drug residues have been successfully implemented to achieve sensitive and specific detection based on AuNRs coupled with recognition molecules containing aptamers, peptides, or antibodies [29,30,31,32].

T-Hg^2+^-T coordination has been proven to be a stable mismatch structure, meaning it is also an ideal molecular recognition element for the specific detection of different mercury species owing to [33,34]. Furthermore, the binding affinity of mercury species to thymine-rich (T-rich) deoxyribonucleic acid (DNA) follows the order MeHg (≈EtHg) > inorganic mercury (Hg^2+^) > other metal ions [35]. Motivated by the aforementioned studies, we expected to perform the analysis of mercury species using a T-rich DNA aptamer combined with AuNRs. However, the AuNR-based biosensor in our previous work had relatively poor sensitivity (2–10 ppm), despite possessing good specificity [36]. Therefore, we develop here a novel colorimetric method by designing a specific sequence, namely a T-richer DNA aptamer, as the recognition probe (RP) and poly (diallyldimethyl ammonium chloride) (PDDAC)-functionalized AuNRs as the signal probe (SP) to recognize and discriminate Hg^2+^ and MeHg, with a view to providing a simpler and more reliable technical reference for the on-site rapid screening and detection of mercury species.

## 2. Results and Discussion

### 2.1. The Experimental Principle for Colorimetric Determination of Hg^2+^ and MeHg

DNA is the major component of chromosomes and the main genetic material; it consists of four nitrogenous bases: adenine (A), thymine (T), guanine (G), and cytosine (C). Thanks to its strict complementary base pairing, specific molecular recognition, and unique self-assembly capability, DNA has extremely broad applications in biosensing analysis, such as for proteins, viruses, metal ions, pathogens, and pesticide residues [37,38,39,40]. Among them, T-rich DNA is deemed the core recognition element in the design of mercury biosensors, as it forms a “T-Hg^2+^-T” mismatched structure with mercury ions, achieving specific discrimination between Hg^2+^ and MeHg [33,34,41,42]. H However, the binding affinity of T-rich DNA with mercury ions is influenced by the position and number of T bases, as well as mercury species [43,44,45]. Furthermore, sulfur-containing groups like a thiol group (-SH) could interact with AuNRs via gold−sulfur (Au-S) covalent bonding, thereby firmly anchoring DNA probes on the surface of AuNRs [46]. Based on the above theories, a T-rich H_T9_ DNA aptamer that was labeled with -SH on its 5′-end was designed as the RP to construct a colorimetric biosensor for the specific determination of Hg^2+^ and MeHg, with PDDAC-coated AuNRs used as the SP.

**Scheme 1:**
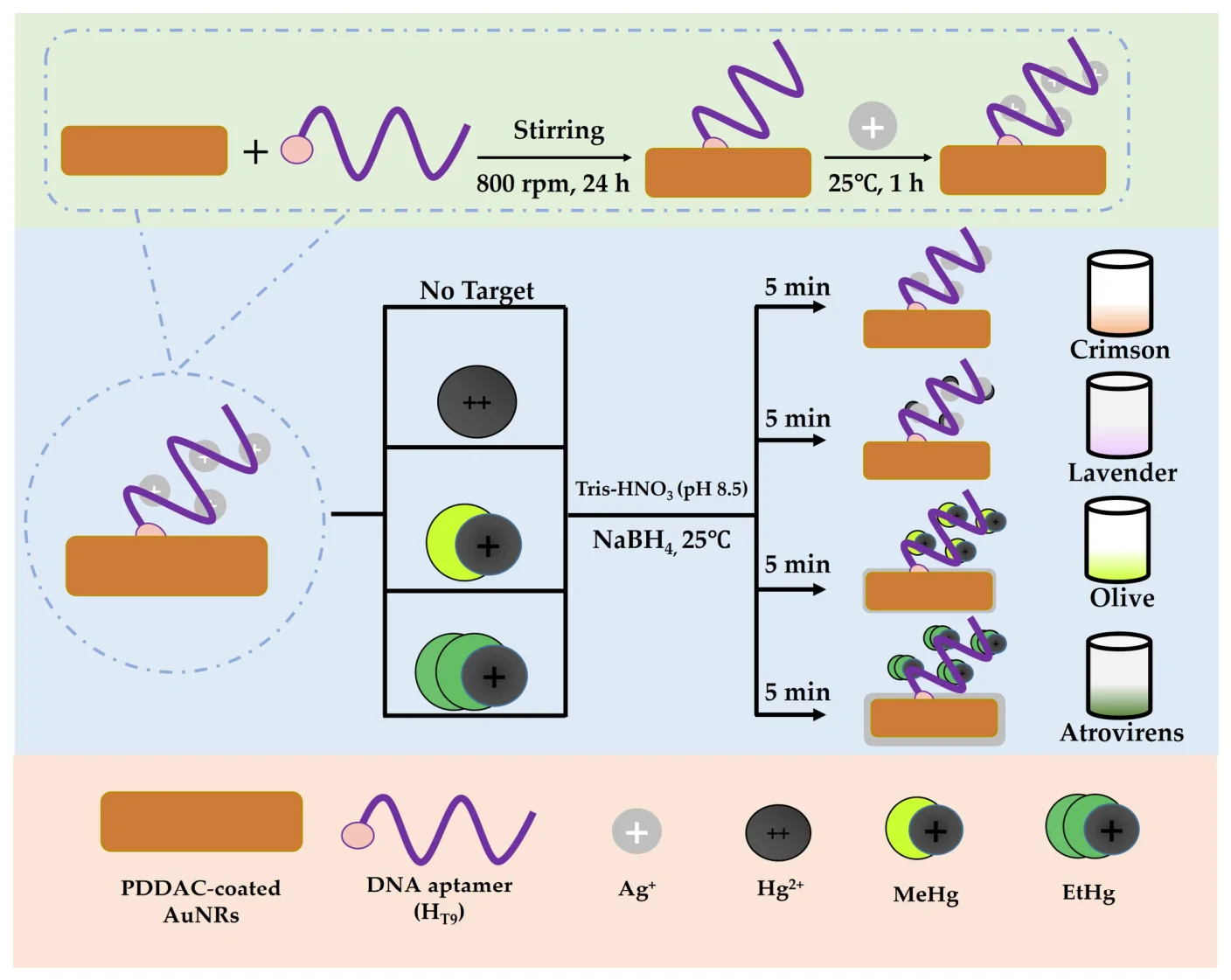
Schematic diagram of colorimetric determination of Hg^2+^ and MeHg.

From Scheme 1, while there were no mercury species, the PDDAC-coated AuNRs maintained the same aspect ratio (AR), and the final reaction solution displayed the same crimson color. In the presence of Hg^2+^, both this and Ag^+^ were together reduced into forming Ag^0^/Hg^0^ amalgam nanoparticles (ANPs) that were bound on the H_T9_ DNA aptamer, which resulted in the final reaction solution being a lighter color (lavender) than the above phenomenon, though the AR of the PDDAC-coated AuNRs was not increased. Nevertheless, the existence of MeHg or EtHg is also different from the two situations mentioned above. Because of the stronger binding ability, MeHg or EtHg showed priority binding to the H_T9_ DNA aptamer over PDDAC-coated AuNRs. After reduction by NaBH_4_, only Ag^0^ NPs were deposited on the surface of PDDAC-coated AuNRs, but MeHg or EtHg still maintained an ionic state. The difference is that PDDAC-coated AuNRs had a slight change in AR in the MeHg-containing final solution, which manifested as a shallower olive color.

### 2.2. Feasibility Analysis and Characterization of the Proposed Method

**Figure 1:**
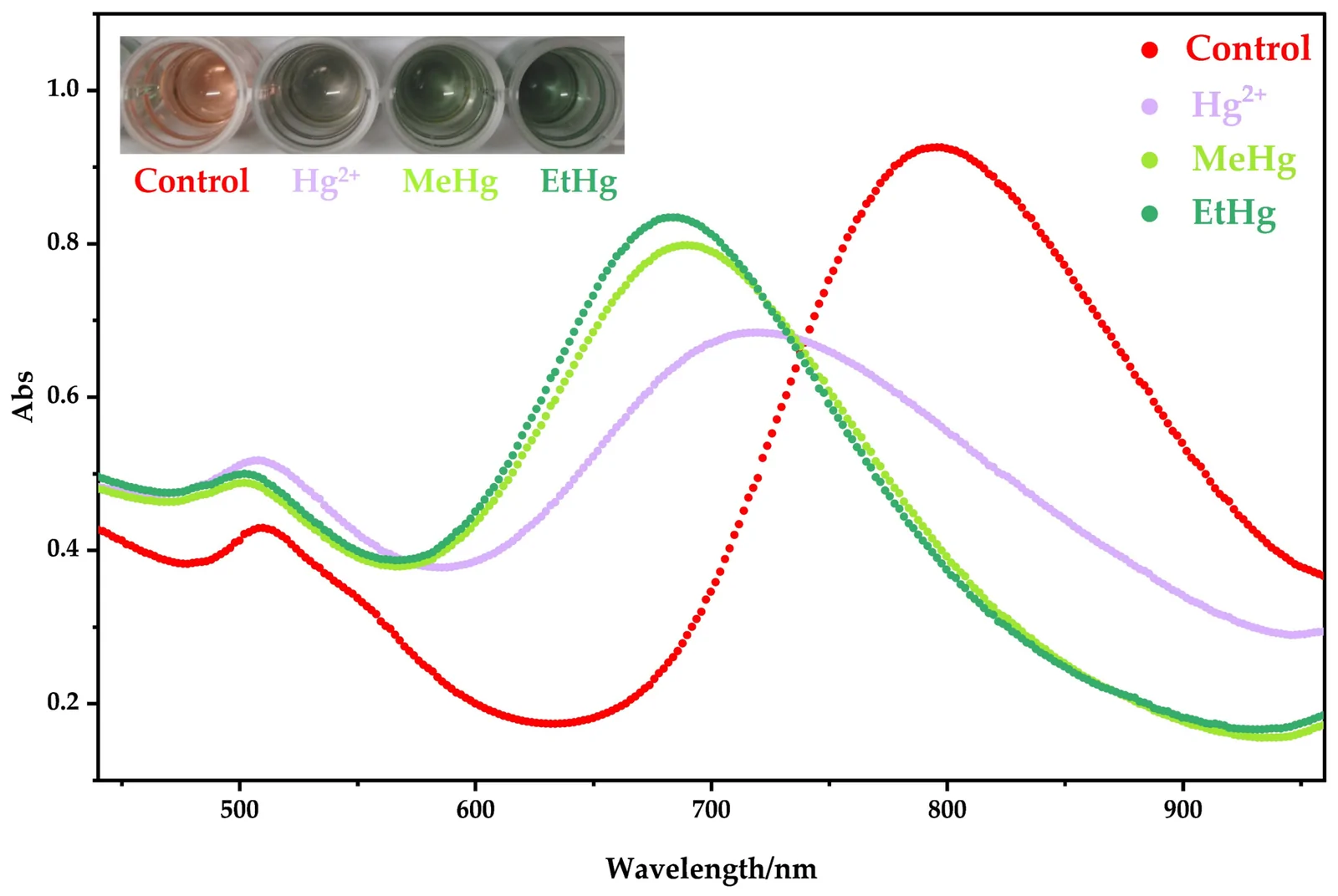
Feasibility analysis for colorimetric determination of Hg^2+^ and MeHg by measuring UV−visible absorption spectra and visual images in our assay system.

To confirm the hypothesis and feasibility of our proposed method, colorimetric analyses, including visual images and UV−visible absorption spectra of the assay system, were first implemented, as shown in Figure 1. With the T-rich H_T9_ DNA aptamer used as the RP, a crimson color was observed in the control group (namely without any mercury species), which was similar to the bare PDDAC-coated AuNRs, and the characteristic absorption peak of the assay system was at a wavelength of 796 nm. When Hg^2+^ existed, the characteristic absorption peak was blue-shifted to 720 nm, and the color of the final reaction solution transformed from crimson to lavender, indicating that the deposit Ag^0^/Hg^0^ ANPs had grown on the T-rich H_T9_ DNA aptamer rather than the PDDAC-coated AuNRs. However, in the presence of MeHg, the final reaction solution depicted a completely different olive color as a result of the AR of the PDDAC-coated AuNRs being changed by the Ag^0^ NP surface depositing layer; its characteristic absorption peak was more blue-shifted to 690 nm. In contrast, a darker atrovirens color was reflected in the EtHg-containing final reaction solution, whose characteristic absorption peak was the most blue-shifted to 684 nm.

An interesting phenomenon was observed: the characteristic absorption peak of the reaction system was blue-shifted by the addition of Hg^2+^. This shift in the absorption spectra or characteristic absorption peak was influenced by various factors, including humidity variations [47]. One possible reason for this is that the formed deposit Ag^0^/Hg^0^ ANPs were surrounded by their local system ambient medium with negative optical nonlinearity [48]. In particular, the Ag^0^/Hg^0^ ANPs might cause an obvious attenuation of the surface plasmon resonance effect on AuNRs, thereby achieving Hg^2+^-specific detection [49].

**Figure 2:**
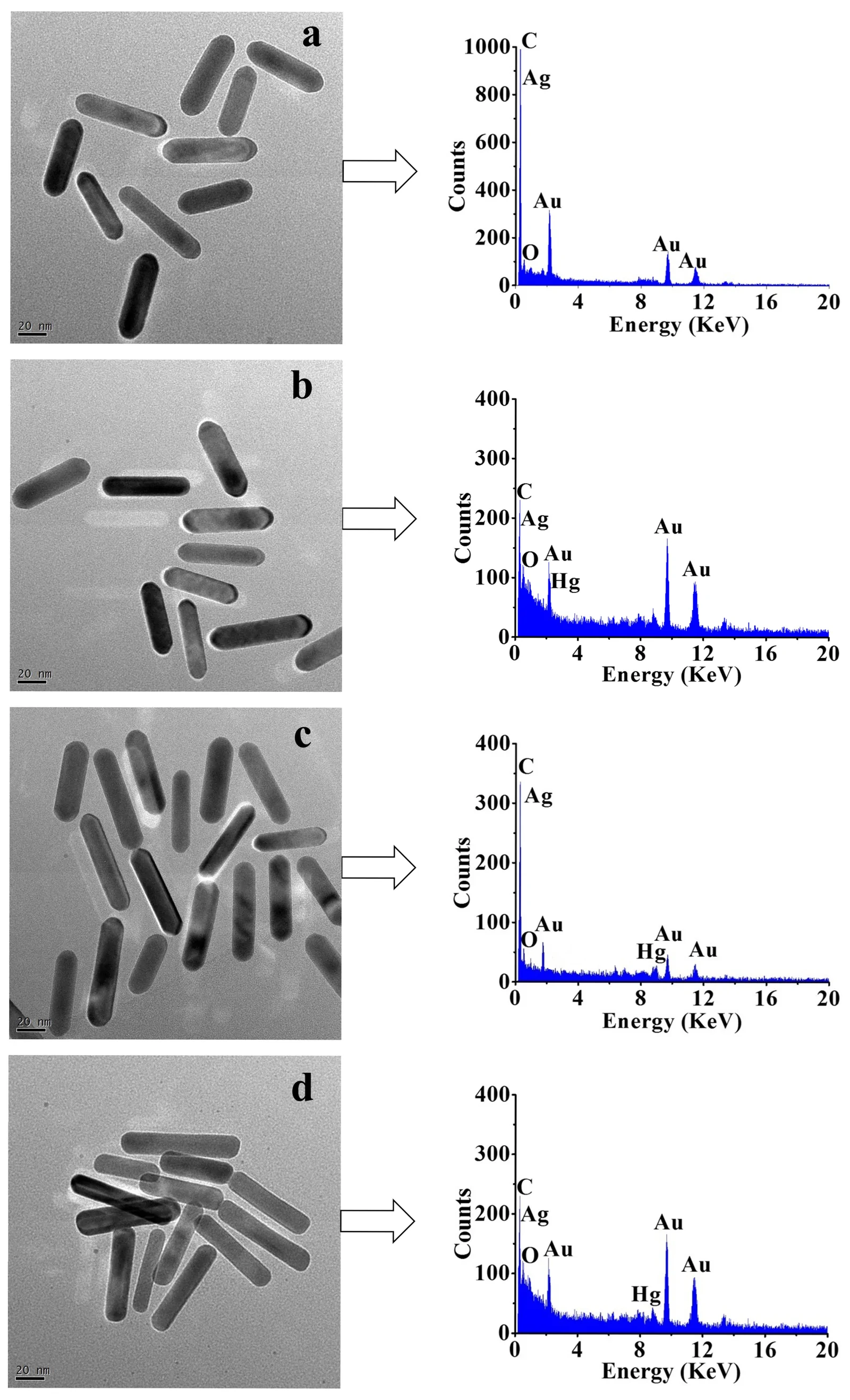
TEM images and EDX analysis of AuNRs in this assay system with control (**a**), Hg^2+^ (**b**), MeHg (**c**) and EtHg (**d**).

To further validate the aforementioned inference, the TEM images and EDX were characterized, as displayed in Figure 2. The PDDAC-coated AuNRs had an average AR of about 3.44 in the control group (Figure S1), and this had evidently not changed (3.52) with the addition of Hg^2+^, demonstrating that the majority of Hg^2+^ was bound with the T-rich H_T9_ DNA aptamer. A small proportion of Hg elements can be detected in the EDX analysis shown in Figure 2c,d, a possible reason for which is that the PDDAC-coated AuNRs adsorbed free Hg^2+^ in the solution. In the presence of MeHg or EtHg, larger ARs were observed for both (>3.90), and the EDX analysis determined plenty of Hg elements, suggesting the preferential binding ability of MeHg or EtHg to the T-rich H_T9_ DNA aptamer.

### 2.3. Optimization of Several Key Experimental Conditions

There are many factors that can influence the specific analysis of mercury species based on the T-rich H_T9_ DNA aptamer-scaffolded biosensor, but several key experimental conditions should be given priority for optimization, such as the reducing agent and its concentration, the pH of Tris-HNO_3_ buffer solution, and AuNRs.

In our design, outlined above, Ag^+^ and Hg^2+^ were reduced by a reducing agent to form Ag^0^/Hg^0^ ANPs; it is crucial to choose an appropriate reducing agent to achieve the best reducing effect. Common reducing agents like TC, NaBH_4_, and AA were set as candidates to compare the effect of reducing agents on the specific recognition for mercury species. Utilizing either TC or AA, as observed in Figure S2, there was not an obvious discrimination between the control group and MeHg-containing group in the UV–visible absorption spectra or visual image, which had nearly overlapped absorption curves (crimson, olive, and atrovirens curves) and a similar visual color. Only when the reducing agent was NaBH_4_ was it just enough to recognize mercury species and meanwhile clearly distinguish Hg^2+^ and MeHg from the perspective of the UV–visible absorption spectra and their characteristic absorption peak, as well as the color of the final reaction solution observed using the naked eye. This could be because, compared with NaBH_4_, the TC or AA possessed poorer stability and a weaker combining capacity to AuNRs in this biosensing system [50,51]. NaBH_4_ was thus regarded as the optimal reducing agent and applied to the following study.

The reducing agent’s concentration also became an important parameter. To determine the suitable concentration, three different concentration points, 70 mM, 85 mM, and 100 mM, of NaBH_4_ were further discussed and explored (Figure S3). The results showed that 70 mM and 100 mM of NaBH_4_ gave rise to the overlap of a characteristic absorption peak and similar colors in the final reaction solution, indicating that an excessively low or excessively high concentration of NaBH_4_ failed to fully excite the reducing performance, including stability and combining capacity. After overall consideration of the color of the four final solutions to the UV–visible absorption spectra at wavelengths ranging from 450 nm to 950 nm, 85 mM of NaBH_4_ was considered to be the optimal concentration.

The pH of Tris-HNO_3_ buffer solution was another key physicochemical factor that could affect the charge state of metal ions, aggregation state of NPs, and specific binding to Hg^2+^ on AuNRs. It even transformed the mercury species in the reaction system [21]. In other words, the UV–visible absorption spectra, characteristic absorption peak, and the color of the final reaction solution might be influenced by the pH of the reaction system. Therefore, we then optimized the pH of the Tris-HNO_3_ buffer solution by setting three pH gradients between 7.5 and 9.5, as depicted in Figure S4. Under alkaline conditions, interestingly, only the control group and Hg^2+^-containing group were obviously affected by the pH of the Tris-HNO_3_ buffer solution. Moreover, the UV–visible absorption spectral curve in the final solution of the four groups drew increasingly close to overlapping as the pH increasing from 8.5 to 9.5. Indeed, the mercury species was also discriminated through UV–visible absorption spectra and visual images at both pH 7.5 and pH 8.5, but the latter was obviously the better option thanks to the slight difference in the UV–visible absorption spectra. Hence, 8.5 was the optimal pH of Tris-HNO_3_ buffer solution.

As is well known, CTAB-coated AuNRs are cytotoxic and erratic, so they require functionalization to improve biocompatibility and optical stability [52]. The surface electronegativity (zeta potential) of AuNRs is mediated by altering various surface modifications to enhance system performance [53]. Therefore, we further investigated the AuNRs with different surface modifications. Figure S5 presents the zeta potential and cell viability of AuNRs with seven different surface modifications. The results demonstrate that compared with other surface-modified AuNRs, the PDDAC-coated AuNRs had a higher positive charge and greater cell viability, implying better stability and low cytotoxicity.

### 2.4. Effect of the Concentration of H_T9_ DNA Aptamer on Assay System

**Figure 3:**
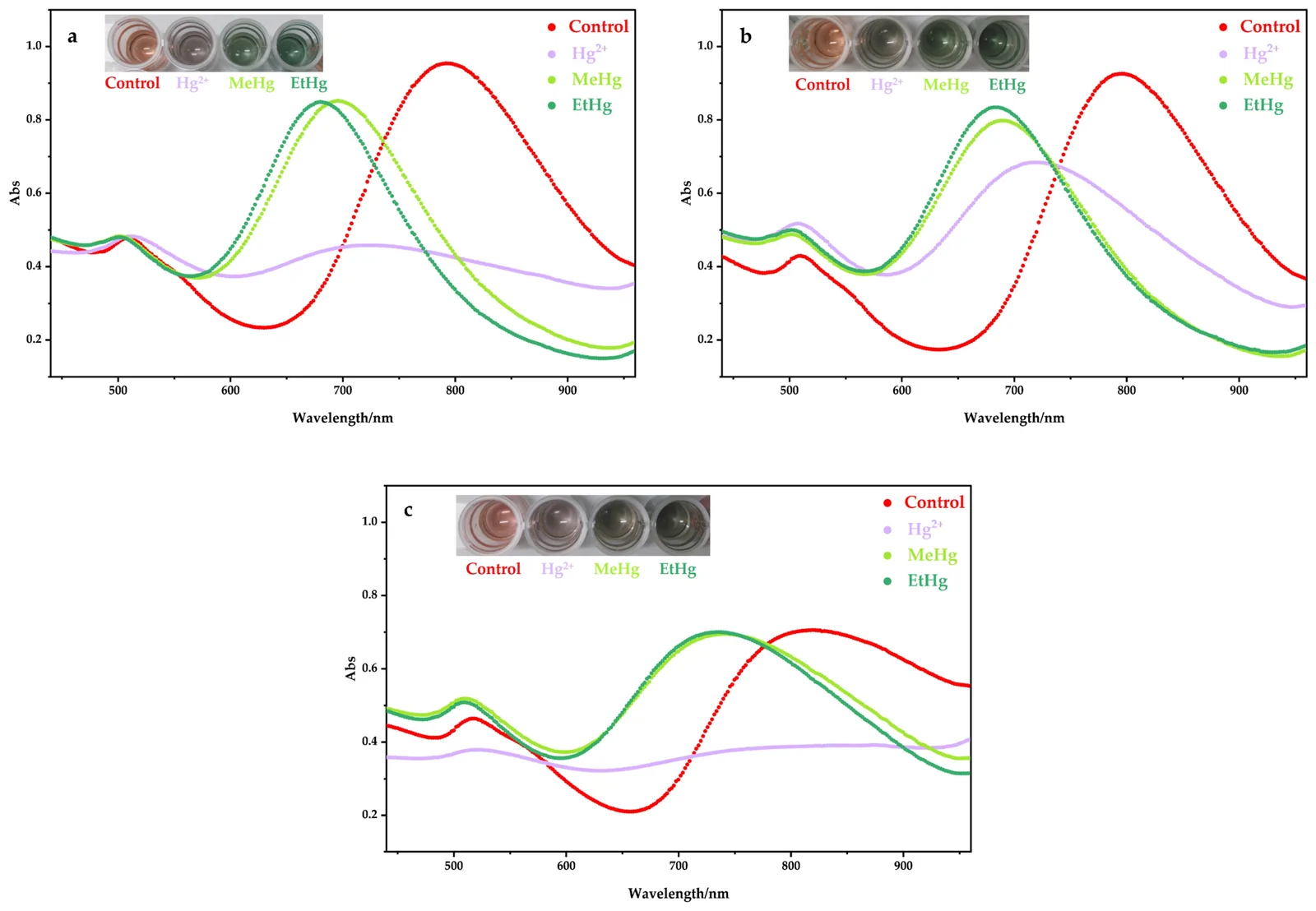
Effect of T-rich H_T9_ DNA aptamer with different concentrations of (**a**) 10 μM, (**b**) 20 μM and (**c**) 30 μM on specific discrimination for mercury species.

Under the optimum experimental conditions described above, the effect of the concentration of the H_T9_ DNA aptamer, ranging from 10 μM to 30 μM, on the analysis system was finally probed. As shown in Figure 3, the UV–visible absorption spectra at the wavelength from 450 nm to 950 nm and visual images were obtained using our proposed method. The UV–visible absorption spectral curves became closer together, except the group with Hg^2+^ as the concentration of the H_T9_ DNA aptamer increased. This may be because a too-low concentration (10 μM) of the H_T9_ DNA aptamer was not sufficient to bind with Hg^2+^, whereas a too-high concentration (30 μM) induced steric hindrance and excess adsorption, interfering with the NP aggregation state induced by Hg^2+^ and then resulting in a similar visual color [54,55]. At a concentration of 20 μM, Hg^2+^ and MeHg were distinctly distinguished by either naked-eye observation or UV–visible absorption spectra. In addition, it would be an ideal phenomenon if an extremely close UV–visible absorption spectral curve and visualized color in only the MeHg- or EtHg-containing groups were observed. Considering overall performance, we eventually selected 20 μM as the optimal concentration of the H_T9_ DNA aptamer.

### 2.5. Selectivity and Specificity Evaluation of the Proposed Method for Detecting Hg^2+^ and MeHg

**Figure 4:**
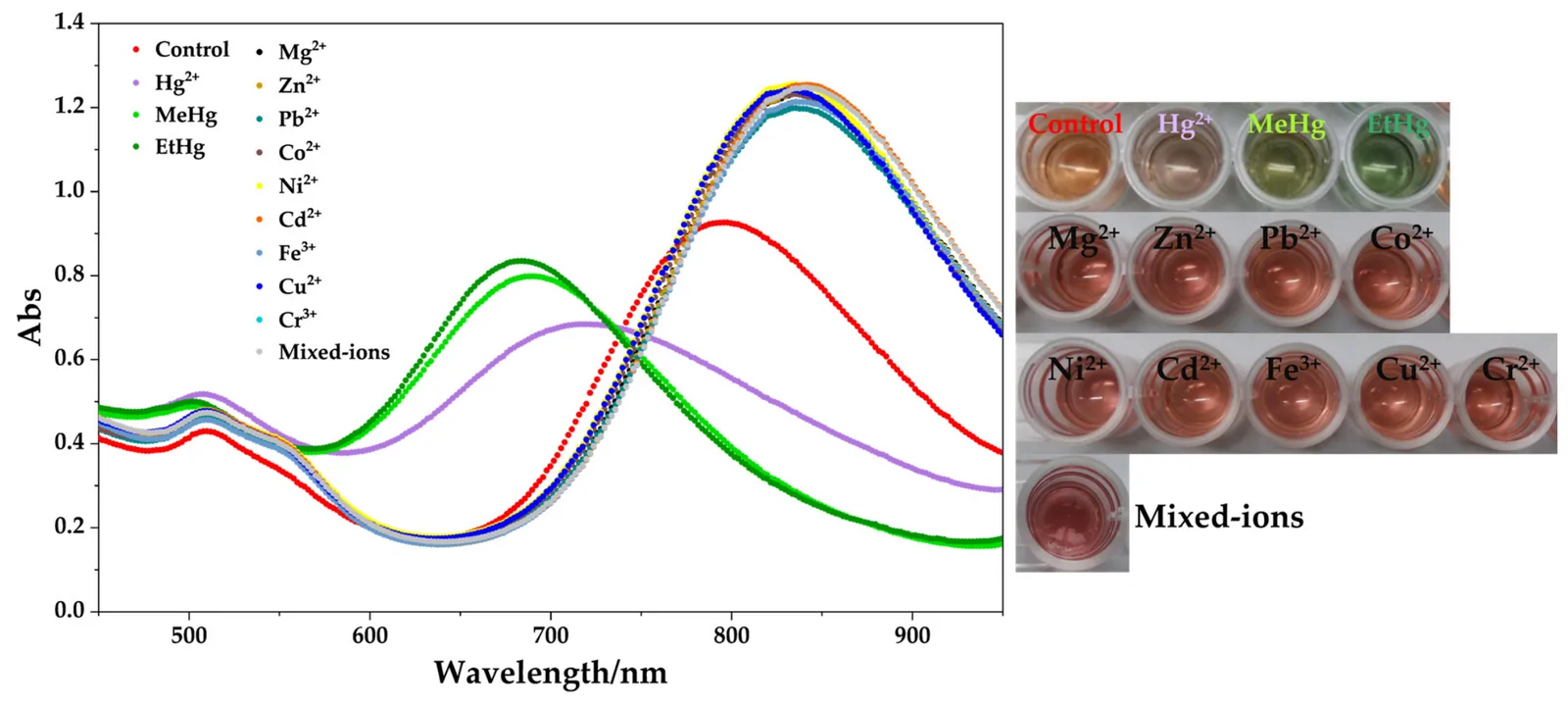
Selectivity and specificity evaluation of the proposed method for detecting Hg^2+^ and MeHg. Mercury species and nine coexisting single metallic ions or mixed ions were measured by scanning UV−visible absorption spectra (**left**) and recording their visual images (**right**), with H_T9_ as RP.

In real samples, especially those with complex matrices, some common metallic ions, including Mg^2+^, Zn^2+^, Pb^2+^, Co^2+^, Ni^2+^, Cd^2+^, Fe^3+^, Cu^2+^, and Cr^3+^, are known to coexist. Using the proposed method, under the above optimal experimental conditions, these coexisting metallic ions might interfere with the assay of mercury species. To assess their impact on the mercury-biosensing method, these nine coexisting metallic ions (at 100 times the concentration of mercury species) were also implemented by the same method and procedure mentioned above. Figure 4 showed that there were similar UV−visible absorption spectra for these nine coexisting metallic ions, whose characteristic absorption peaks were blue-shifted from 796 nm to 836 nm, and they had the same crimson color. This result demonstrates that our proposed method possessed excellent selectivity to mercury species and had good specificity to Hg^2+^ and OrHg.

### 2.6. Analytical Performance Evaluation of the Proposed Method for Detecting Hg^2+^ and MeHg

**Figure 5:**
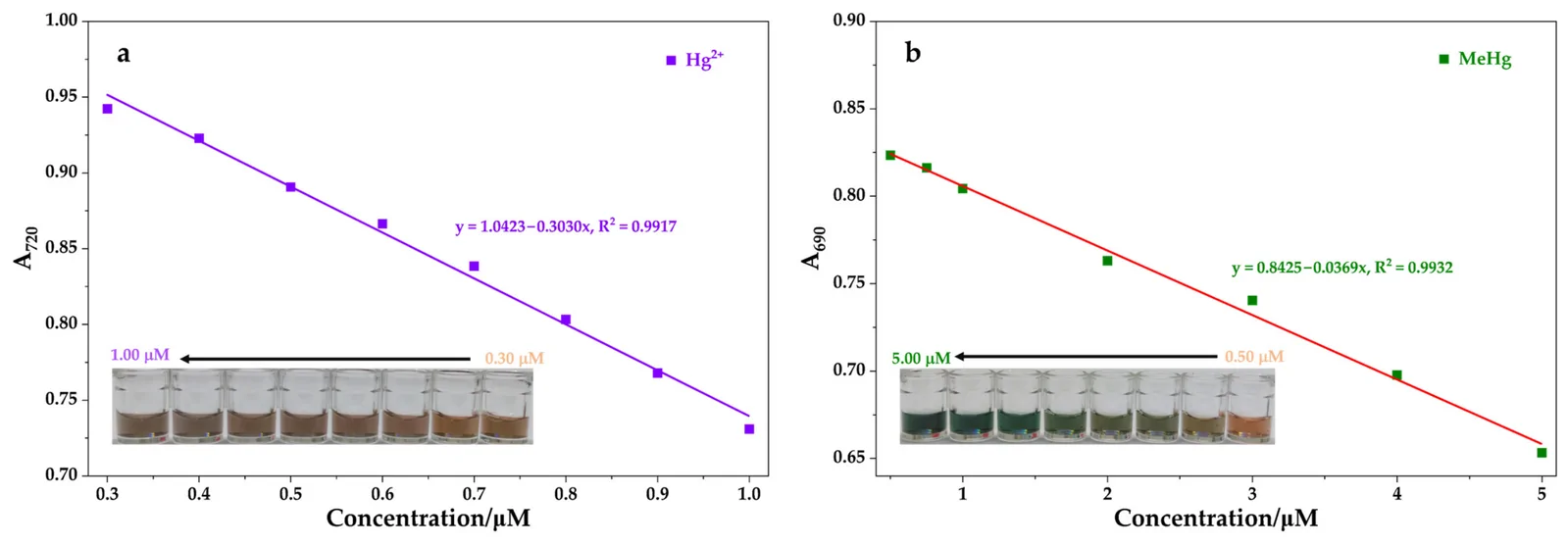
Visual images and calibration curves for detecting Hg^2+^ (**a**) ranging from 0.30 μM to 1.00 μM and MeHg (**b**) varying from 0.50 μM to 5.00 μM.

For realizing optimum analytical performance, under the same procedures and optimal conditions above, the absorbance of UV−visible characteristic absorption peaks at wavelength of 720 nm (A_720_, Hg^2+^) and 690 nm (A_690_, MeHg) as well as corresponding visual images were collected with increase in concentrations of Hg^2+^ or MeHg (Figure 5). Their UV−visible absorption spectra (Figure S6) and the relationship between the peak shift (Δλ) and concentration (Figure S7) indicated that the addition of different concentrations of Hg^2+^ slightly altered the UV−visible characteristic absorption peak, which was still influenced by MeHg ranging from 0.5 μM to 3.0 μM with a calibration curve of Δλ = 66.32 + 26.814 × C (R^2^ = 0.9919). The A_720_ (or A_690_) decreased and the color of the final reaction solution gradually deepened as the concentration of Hg^2+^ or MeHg increased. This may be because the higher the mercury concentration, the larger the Ag^0^/Hg^0^ ANPs or the thicker Ag^0^ NPs deposited on the surface of PDDAC-coated AuNRs, which caused greater interference with the absorbance of the system solution and therefore decreased the characteristic-peak absorbance. Linear equations further implied a good linear relationship between A_720_ (or A_690_) and their concentrations in the range of 0.30–1.00 μM and 0.50–5.00 μM for Hg^2+^ and MeHg, respectively. In other words, the visual LOD of this method was 0.30 μM (for Hg^2+^) and 0.50 μM (for MeHg). Specifically speaking, the regression equations by linear fitting were obtained as following: A_720_ = 1.0423 − 0.3030 × C (R^2^ = 0.9917) for Hg^2+^; A_690_ = 0.8425 − 0.0369 × C (R^2^ = 0.9932) for MeHg, where C representing the concentration of Hg^2+^ or MeHg with the unit of μM. Their theoretical LOD (3σ/S) was calculated to be 0.071 μM and 0.12 μM for Hg^2+^ and MeHg, respectively. The above results outline the good analytical sensitivity of the colorimetric biosensing method we exploited for Hg^2+^ and MeHg detection utilizing T-rich H_T9_ DNA aptamer as the RP and PDDAC-coated AuNRs as the SP.

According to the latest Chinese National Food Safety Standard, the limits for the maximum levels of mercury are 0.5–1.7 mg/kg (namely 2.3–7.9 μM) [56] for MeHg in aquatic products and 0.001 mg/L (namely 5.0 nM) [57] for Hg^2+^ in drinking water. Thus, our method displayed a good visual LOD for determining aquatic products, and it was also helpful as a semi-quantitative on-site screening tool for determining Hg^2+^ in drinking water. Furthermore, the sensitivity of our proposed aptamer-based colorimetric method was lower than the majority of the other reported sensing methods and instrumental techniques, indicating that there is still much room for improvement for our method (Table 1).

### 2.7. Determination of Actual Seawater Sample

With good sensitivity and selectivity in the Hg^2+^ or MeHg standard solution, the proposed colorimetric biosensing method was employed to determine actual seawater samples collected from coastal water of Longhai, Fujian, China, to assess its practical reliability. The sample spiked with different concentrations of Hg^2+^ or MeHg, and it was also detected using the same method for Rec. As displayed in Table 2, the Hg^2+^ in the actual seawater sample can be specifically detected with naked-eye observation or UV−visible absorption spectra by using the H_T9_ DNA aptamer with a Rec. of 104–107% and an RSD (n = 6) ≤ 8%. Additionally, the MeHg in the same actual seawater sample can also be detected accurately with visual images or UV−visible absorption spectra by using H_T9_ as an SP with a Rec. of 98–106% and an RSD (n = 6) ≤ 7%. Through capillary electrophoresis integrated with ICP-MS (CE-ICP-MS), all actual seawater samples were validated again [38]. This shows our method to be reliable and practical, enabling its application to specifically instrument-free colorimetric biosensing detection of Hg^2+^ and MeHg in seawater.

## 3. Materials and Methods

### 3.1. Experimental Reagents

The sequences of DNA aptamer used in this work were synthesized and verified by Sangon Biotech Co., Ltd. (Shanghai, China). Tris-(hydroxymethyl) aminomethane (Tris) was provided by Merck (Darmstadt, Germany). Silver nitrate (AgNO_3_), trisodium citrate (TC), and cetyltrimethylammonium bromide (CTAB) was purchased from Sigma-Aldrich Company (Shanghai, China). PDDAC, polyethyleneimine (PEI), polystyrene sulfonate (PSS), and poly (ethylene glycol) (PEG) were obtained from Shanghai Macklin Biochemical Technology Co., Ltd. (Shanghai, China). Methyl-mercuric chloride (MeHgCl) and ethyl-mercuric chloride (EtHgCl) were purchased from Dr. Ehrenstorfer GmbH (Augsburg, Germany). Chloroauric acid trihydrate (HAuCl_4_·3H_2_O) and mercury nitrate monohydrate (Hg(NO_3_)_2_·H_2_O) were produced by J&K Scientific Group (Beijing, China). Nitric acid (HNO_3_) was provided by Xilong Chemical Co., Ltd. (Guangzhou, China). Sodium borohydride (NaBH_4_) and L (+)-ascorbic acid (AA) were purchased from Sinopharm Chemical Reagent Co., Ltd. (Shanghai, China). RPMI1640 cell culture medium and fetal bovine serum (FBS) were purchased from Gibco (Logan, UT, USA). Cell viability was assessed using a Cell Counting Kit-8 (CCK-8) from Dojindo (Kumamoto-ken, Japan).

We ensured that the concentrations of each DNA aptamer were accurately quantified by optical density (OD) at a wavelength of 260 nm (OD_260_) based on the absorption coefficient. NaBH_4_ solution also must be freshly prepared prior to use. Tris solution was adjusted by HNO_3_ solution (1.0 M) to prepare Tris−HNO_3_ buffer solution (10 mM) at different pH values. All reagents were of commercially available analytical reagent grade at least. Ultrapure water (18.2 MΩ·cm) was used throughout the whole experiment to eliminate the interference of impurity in water.

### 3.2. Experimental Instruments

The absorbance and UV–vis absorption spectroscopy were recorded by Tecan’s Infinite M200 PRO multifunctional microplate reader (Tecan Group Ltd., Männedorf, Switzerland). Transmission electron microscope (TEM) images and energy-dispersive X-ray (EDX) spectra analysis were measured by FEI Tecnai G2 F20 U-TWIN (FEI Company, Portland, OR, USA). Mass spectrometric (MS) analysis was performed on Thermo Fisher TSQ Altis mass spectrometry (Thermo Fisher Scientific Inc., Waltham, MA, USA). The concentrations of three species of mercury ions were confirmed by Agilent 7800 ICP-MS (Agilent Technologies Inc., Santa Clara, CA, USA). Ultrapure water was produced using a sterile Millipore Milli-Q ultrapure water system (Sigma-Aldrich Corporation, Bedford, MA, USA). The zeta potential was analyzed using the Malvern Panalytical Zetasizer Nano ZS90 analyzer (Malvern, UK). The pH of Tris-HNO_3_ buffer solution was measured using Ohaus Starter 2100/E acidometer (Ohaus Corporation, Parsippany, NJ, USA). The low-temperature high-speed centrifuge and single-channel/multi-channel pipettes were purchased from Eppendorf AG, Hamburg, Germany.

### 3.3. Experimental Methods

#### 3.3.1. Synthesis of CTAB-Coated AuNRs

A seed-mediated growth was employed as the method to synthesize CTAB-coated AuNRs depending on the order from CTAB-capped Au seeds to maturation [62]. More concretely, 12 mL of CTAB solution (100 mM) was firstly added to a 50 mL glass bottle with 300 μL of HAuCl_4_ solution (10 mM). After gentle mixing, 720 μL of ice-cold NaBH_4_ solution (freshly prepared, 10 mM) was then added rapidly into the above reaction solution and repeatedly stirred for 3 min. CTAB-capped Au seeds were successfully prepared when brownish yellow appeared in the solution. In terms of the seed solution, it was necessary to incubate it for 2 h at room temperature for further use.

Subsequently, the precursor solution for AuNR growth consisting of 48 mL of CTAB solution (100 mM), 2.4 mL of HAuCl_4_ solution (10 mM), and 500 μL of AgNO_3_ solution (10 mM) was vortexed thoroughly. After using 1.0 mL of HCl solution (1.0 M) to adjust the pH of the above mixture to approximately 2.0, 400 μL of AA solution (0.1 M) was introduced to form the growth solution of AuNRs. To obtain CTAB-coated AuNRs, 1.0 mL of the above prepared seed solution was added to the prepared growth solution, accompanied by gentle mixing and incubation at room temperature for 24 h to full maturation. Ultimately, the as-synthesized CTAB-coated AuNR solution was concentrated 10 times and centrifugated at 6000 rpm for 20 min, and the precipitate was dispersed in aqueous solution while used.

#### 3.3.2. Construction of AuNRs-Based Biosensor

To prepare the AuNR-based biosensors, to begin with, 15 mL of the above precipitate of CTAB-coated AuNR solution was dispersed in 15 mL of PSS solution (2.0 mM). After stirring for 1 h, the mixture was centrifuged at 10,000 rpm for 10 min and the precipitate was resuspended using 1.2 mL of ultrapure water to form PSS-coated AuNRs. On this basis, PDDAC-coated AuNRs were obtained similarly to treatment with PSS-coated AuNRs. Then, 20 μL of PDDAC-coated AuNR solution was added to 20 μL of T-rich DNA aptamer solution (20 μM of H_T9_, with its sequences: 5′-HS-TTGTTCTTTGTTAAAAATTCTTTGTTCTT-3′) with gentle mixing. The final solution was stirred at 800 rpm for 24 h to bind H_T9_ DNA aptamer on the surface of AuNRs and centrifuged at 5000 rpm for 20 min to remove 30 μL of supernatants, resulting in the construction of the AuNR-based biosensor.

#### 3.3.3. Specific Colorimetric Determination of Hg^2+^ and MeHg

A total of 10 μL of AgNO_3_ solution (1.0 mM) was added to a 200 μL centrifuge tube, which contained the solution of the above AuNR-based biosensor. The mixture was incubated at 25 °C for 1 h. Then, 10 μL of various concentrations of Hg^2+^ or MeHg standard solution was added to interact specifically with the AuNR-based biosensor. After the addition of 100 μL of Tris-HNO_3_ buffer (pH 8.5) and 10 μL of NaBH_4_ solution (85 mM) to the above reaction solution, it was stirred gently for 15 s. Prior to observing and recording the color change in the mixture, it was incubated at 25 °C for 5 min without vortex so as to provide a sufficient reducing condition. Finally, the UV–visible absorption spectrum of the final solution was scanned in the range of 450–950 nm.

#### 3.3.4. Cell Viability Analysis

Human cervical cancer cells (Hela cells) were used to confirm the cytotoxicity of AuNRs with different surface modifications. Hela cells were initially cultured with RPMI 1640 cell culture medium containing 10% heat-inactivated FBS. The cell seeding density of Hela cells was 1 × 10^4^ cells/mL. Hela cells were washed twice with PBS solution and digested with 0.25% Trypsin-0.53 mM EDTA solution. Then, 20 μL of different concentrations of AuNR solution was added to RPMI 1640 cell culture medium and co-incubated for 6 h at 37 °C with 5% CO_2_. After adding 10 μL of CCK-8 solution, to evaluate the cell viability, Hela cells continued to be incubated for 4 h in the dark. The absorbance at 450 nm (OD_450_) of the final solution was recorded. Cell viability (%) = [OD_450_ (AuNRs) − OD_450_ (blank control) / (OD_450_ (negative control) − OD_450_ (blank control)] × 100%.

#### 3.3.5. Analysis of Hg^2+^ and MeHg in Actual Seawater Sample

A sample of real seawater from the coastal water of Longhai, Fujian, China, was pretreated according to the relevant references [63,64]. Briefly, the seawater sample was further filtered using the filter membrane (0.22 µm) to remove residues and macromolecular substances. At the same time, the filtrate was collected and directly detected using the above method to obtain the Hg^2+^ content. Due to the concentration of MeHg in the seawater sample being much lower than Hg^2+^, the accuracy of the measured MeHg might be affected. Therefore, before the formal analysis of MeHg in the seawater sample, which should be handled through adding an appropriate amount of AA (70 mM) to remove Hg^2+^ by transforming it into an elemental state (Hg^0^), nitrogen (N_2_) was then introduced for 5 min to remove Hg^0^. The seawater samples were also spiked with different concentrations of Hg^2+^ or MeHg and detected using the method proposed in Section 3.3.3 to obtain Rec. and RSD.

## 4. Conclusions

In this work, a semi-quantitative on-site screening method based on the T-rich H_T9_ DNA aptamer being used as the RP and PDDAC-coated AuNRs as the SP was established for the specific discrimination of Hg^2+^ and MeHg. Utilizing the difference in binding affinity between Hg^2+^ and MeHg for the H_T9_ DNA aptamer labeled with -SH, PDDAC-coated AuNRs were stably anchored using the H_T9_ DNA aptamer via Au-S covalent bonding or deposited by forming a Ag^0^ NP layer, thereby altering the AR and eventually resulting in different visual colors and UV−visible characteristic absorption peaks in the final reaction solution. The proposed strategy resulted in a good linear relationship between A_720_ (or A_690_) and their concentrations in the range of 0.30–1.00 μM and 0.50–5.00 μM for Hg^2+^ and MeHg, respectively, with a low visual LOD of 0.30 μM for Hg^2+^ and 0.50 μM for MeHg. The theoretical LODs (3σ/S) were calculated to be 0.071 μM for Hg^2+^ and 0.12 μM for MeHg. This method was successfully applied to the colorimetric detection of Hg^2+^ and MeHg in actual seawater samples. We expect this method to provide a useful technical reference for the determination of Hg^2+^ and MeHg in drinking water, aquatic products, and food, especially in the case of accident-level contamination.

## Figures and Tables

**Table 1:** Comparison of analytical performance of our method versus other reported methods.

Methods	Selectivity	Analytical Performance			References
		LOD (μM)	LOQ (nM)	Linear Range (μM)	
Colorimetric	Hg^2+^; OrHg	2.0–10.0	-	0.0–100.0	[36]
Fluorescent	Hg^2+^	0.5	12.0	0.5–2.5	[58]
Fluorescent	OrHg	0.05	5.0	0.05–5.0	[44]
Electrochemical	Hg^2+^	0.01	0.01	1.0 × 10^−6^–0.01	[59]
Cold vapor atomic absorption spectroscopy (CVAAS)	Hg^2+^	1.0 × 10^−4^	-	2.0 × 10^−4^–1.2 × 10^−3^	[60]
Single-cell inductively coupled plasma-mass spectrometry (SC-ICP-MS)	Hg^2+^	4.0 × 10^−5^	-	0.05–500	[61]
Colorimetric	Hg^2+^; MeHg	0.30–0.50	-	0.30–5.00	This work

**Table 2:** Analytical results of Hg^2+^ or MeHg in actual seawater sample.

DNA Aptamer		Added Hg^2+^ (μM)	Added MeHg (μM)	Hg^2+^ or MeHg Detection				CE-ICP-MS	
				Naked-Eye Observation	UV-Vis Spectrometry			Concentration (μM)	
					Concentration (μM)	Rec. (%)	RSD (n = 6)	Hg^2+^	MeHg
H_T9_	1	0	0	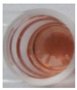	0.21	-	8%	-	-
	2	0.30	0	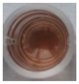	0.42	107	6%	0.36	0
	3	0.50	0	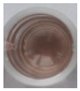	0.66	104	5%	0.61	0
	4	1.00	0	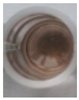	1.25	105	6%	1.18	0
	1	0	0	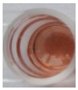	0.13	-	7%	-	-
	2	0	0.50	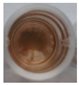	0.47	98	5%	0	0.49
	3	0	1.00	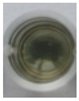	1.19	106	4%	0	1.12
	4	0	3.00	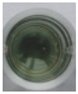	3.04	103	5%	0	3.01
	5 #	0	3.00	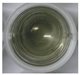	3.16	104	5%	0.17	3.05

- No Hg^2+^ or MeHg detected. # No AA/N_2_ pretreatment.

## Data Availability

The details of the data supporting the reported results are available in the article. Further inquiries can be directed to the corresponding author.

## Supplementary Materials

The following supporting information can be downloaded at https://www.mdpi.com/article/10.3390/molecules31213925/s1: Figure S1: The average aspect ratio (AR) of PDDAC-coated AuNRs with/without mercury species; Figure S2: Optimization of the reducing agent. (a) TC; (b) NaBH_4_; (c) AA; Figure S3: Optimization of the NaBH_4_ concentration. (a) 70 mM; (b) 85 mM; (c) 100 mM; Figure S4: Optimization of the pH in the system solution. (a) pH 7.5; (b) pH 8.5; (c) pH 9.5; Figure S5: The zeta potential and cell viability of AuNRs with seven different surface modifications. Figure S6: The UV-vis absorption spectra of the H_T9_ DNA aptamer-AuNRs composites in the presence of different concentrations of Hg^2+^ or MeHg. Figure S7: The relationship between the peak shift (Δλ) and concentration in the presence of different concentrations of Hg^2+^ or MeHg.

## Abbreviations

The following abbreviations are frequently used in this manuscript:

MeHg	Methylmercury
EtHg	Ethylmercury
T-Hg^2+^-T	Thymine-Hg^2+^-thymine
T-rich	Thymine-rich
AuNRs	Gold nanorods
DNA	Deoxyribonucleic acid
RP	Recognition probe
PDDAC	Poly (diallyldimethyl ammonium chloride)
SP	Signal probe
TC	Trisodium citrate
CTAB	Cetyltrimethylammonium bromide
PEI	Polyethyleneimine
PSS	Polystyrene sulfonate
PEG	Poly (ethylene glycol)
AA	Ascorbic acid
CCK-8	Cell Counting Kit-8
OD	Optical density
TEM	Transmission electron microscope
MS	Mass spectrometric
Rec.	Recovery
RSD	Relative standard deviation
AR	Aspect ratio
NPs	Nanoparticles
ANPs	Amalgam nanoparticles

## References

[1] Lopreside A., Montali L., Wang B., Tassoni A., Ferri M., Calabretta M.M., Michelini E. (2021). Orthogonal paper biosensor for mercury(II) combining bioluminescence and colorimetric smartphone detection. Biosens. Bioelectron..

[2] Peng H., Rong Y., Chen D., Sun R., Huang J., Ding H., Olid C., Yan H. (2023). Anthropogenic activity and millennial climate variability affect Holocene mercury deposition of an alpine wetland near the largest mercury mine in China. Chemosphere.

[3] González-Reguero D., Robas-Mora M., Lobo A.P., Gómez P.A.G. (2023). Bioremediation of environments contaminated with mercury. Present and perspectives. World J. Microbiol. Biotechnol..

[4] Chen Z., He Y. (2026). Mercury contamination in food: Sources, hazards, prevention and detection techniques for mercury speciation analysis: A review. J. Chem. Soc. Pak..

[5] Ray S., Vashishth R., Mukherjee A.G., Gopalakrishnan A.V., Sabina E.P. (2025). Mercury in the environment: Biogeochemical transformation, ecological impacts, human risks, and remediation strategies. Chemosphere.

[6] Santos L.F., Baldo D.Â., Oliveira J.M., Vila M.M.D.C., Balcão V.M. (2025). An environmental “fairytail”: Removal of mercury from water via phage virion-based biosorption. Enzym. Microb. Technol..

[7] Lin H., Wei Y., Huang J., Lu Q., Zhu S., Qin J., Su S., He T. (2025). Acute effects of mercury on brine shrimp: First evidence highlighting the molecular mechanism through integrated transcriptome and metabolomic analysis. Ecotox. Environ. Saf..

[8] Yang K., Wang Y., Lv C. (2025). Applications of protein-based nanomaterials in mercury detection and removal: Advantages and challenges. Environ. Res..

[9] Li Z., Hou T., Long W., Chen B., Li L., Li Y., Zhou H., Zhu Y. (2026). Quantitative determination of mercury in blood-based DL-cysteine-modified direct mercury analyzer method. Front. Bioeng. Biotechnol..

[10] Fioravanti R., Muzzioli L., Maurel E., Palma G., Calabrese G., Angioni A., Rocca C.L., Mantovani A., Pezzana A., Donini L.M. (2025). Bioaccumulation and biomagnification of mercury along the seafood chain in Europe: A systematic review. Foods.

[11] Wang S., Wang F., Lu C., Yang X., Ma S., Liu Y., Liao K., Zhang S., Zhao J., Wang L. (2025). Thiourea-enhanced bifunctional covalent organic framework triboelectric nanosensor for high-efficiency detection and visualization of Hg ions. J. Hazard. Mater..

[12] Trujillo-Cruz E., Fernández L., Carpintero-Salvador N., Ruiz-Urigüen M., Martínez-Mora E.O., Bolaños-Méndez D., Espinoza-Montero P. (2026). Total mercury exposure through canned tuna in oil sold in Quito Ecuador. npj Sci. Food.

[13] Favilli L., Giacomino A., Malandrino M., Inaudi P., Diana A., Abollino O. (2022). Strategies for mercury speciation with single and multi-element approaches by HPLC-ICP-MS. Front. Chem..

[14] Abouelenein D., Henares M., Fuentes A., Fernández-Segovia I., Barat J.M., Loeschner K., Duedahl-Olesen L., Gómez-Gómez M., Griol A., Sloth J.J. (2026). Conventional Chromatographic Techniques and Biosensors for Mercury Speciation in Seafood: A Systematic Review. Foods.

[15] Pennacchio A., Giampaolo F., Piccialli F., Cuomo S., Notomista E., Spinelli M., Amoresano A., Piscitelli A., Giardina P. (2022). A machine learning-enhanced biosensor for mercury detection based on an hydrophobin chimera. Biosens. Bioelectron..

[16] Carter J.A., Barber C.A., Wolle M.M., Gray P.J. (2025). Development and validation of a non-chromatographic method for mercury and methylmercury in finfish using thermal decomposition gold amalgamation atomic absorption spectrophotometry (TDA-AAS) and salting-out assisted liquid–liquid extraction (SALLE). Anal. Bioanal. Chem..

[17] Lim J.W., Kim T.Y., Woo M.A. (2021). Trends in sensor development toward next-generation point-of-care testing for mercury. Biosens. Bioelectron..

[18] Liu J., Chen J., Wang Y., Wu D., Wang X., Wu Y., Li G. (2026). Progress in advanced biosensors for authentication of animal-origin food. Crit. Rev. Food Sci. Nutr..

[19] Wang J., Wang J., Zhou P., Tao H., Wang X., Wu Y. (2020). Oligonucleotide-induced regulation of the oxidase-mimicking activity of octahedral Mn_3_O_4_ nanoparticles for colorimetric detection of heavy metals. Microchim. Acta.

[20] Kant T., Shrivas K., Tejwani A., Tandey K., Sharma A., Gupta S. (2023). Progress in the design of portable colorimetric chemical sensing devices. Nanoscale.

[21] Xie Y. (2018). Colorimetric determination of Hg(II) via the gold amalgam induced deaggregation of gold nanoparticles. Microchim. Acta.

[22] Chen Z., Wang X., Cheng X., Yang W., Wu Y., Fu F. (2018). Specifically and visually detect methyl-mercury and ethyl-mercury in fish sample based on DNA-templated alloy Ag–Au nanoparticles. Anal. Chem..

[23] Zheng J., Cheng X., Zhang H., Bai X., Ai R., Shao L., Wang J. (2021). Gold nanorods: The most versatile plasmonic nanoparticles. Chem. Rev..

[24] Pandey P.K., Singh P.P., Khatua S., Ranganathan R., Mishra A. (2025). In vitro and in-silico assessment of gaussian curvature-driven internalization kinetics of nanoparticles. ACS Appl. Mater. Interfaces.

[25] Rodrigo J.A., Alieva T., Manzaneda-Gonzalez V., Guerrero-Martinez A. (2024). All-optical trapping and programmable transport of gold nanorods with simultaneous orientation and spinning control. ACS Nano.

[26] Nejabat M., Samie A., Ramezani M., Alibolandi M., Abnous K., Taghdisi S.M. (2023). An overview on gold nanorods as versatile nanoparticles in cancer therapy. J. Control. Release.

[27] Kesharwani P., Ma R., Sang L., Fatima M., Sheikh A., Abourehab M.A., Gupta N., Chen Z., Zhou Y. (2023). Gold nanoparticles and gold nanorods in the landscape of cancer therapy. Mol. Cancer.

[28] Sun I.C., Dumani D.S., Emelianov S.Y. (2021). Applications of the photocatalytic and photoacoustic properties of gold nanorods in contrast-enhanced ultrasound and photoacoustic imaging. ACS Nano.

[29] Yu W., Li Q., Ren J., Feng K., Gong J., Li Z., Zhang J., Liu X., Xu Z., Yang L. (2024). A sensor platform based on SERS detection/janus textile for sweat glucose and lactate analysis toward portable monitoring of wellness status. Biosens. Bioelectron..

[30] Guo Z., Zhang M., Zhang H., Ren X., Xiao Y., Sun W., Wang Y., Liu S., Huang J. (2024). Effective multicolor visual biosensor for ochratoxin A detection enabled by DNAzyme catalysis and gold nanorod etching. Microchim. Acta.

[31] Li Z., Lin Z., Chen L., Lin Y., Luo F., Lin C., Wang J., Qiu B., Lin Z. (2025). Electrochemiluminescence sensor for organophosphorus pesticides based on the regulation of resonance energy transfer between negative charged gold nanorods and Ru(bpy)_3_^2^. Talanta.

[32] Li M., Wei G., Liang Z., Wang W., Wen C., Sun L., Lin X.C. (2025). Porous boron nitride-coupled gold nanorods enable highly sensitive SERS detection of tetracycline hydrochloride. Microchim. Acta.

[33] Ono A., Togashi H. (2004). Highly selective oligonucleotide-based sensor for mercury(II) in aqueous solutions. Angew. Chem. Int. Ed. Engl..

[34] Deng L., Li Y., Yan X., Xiao J., Ma C., Zheng J., Liu S., Yang R. (2015). Ultrasensitive and highly selective detection of bioaccumulation of methyl-mercury in fish samples via Ag^0^/Hg^0^ amalgamation. Anal. Chem..

[35] Li Y., Jiang Y., Yan X.P. (2006). Probing mercury species-DNA interactions by capillary electrophoresis with on-line electrothermal atomic absorption spectrometric detection. Anal. Chem..

[36] Chen Z., Li P., Cheng X., Yang W., Wu Y., Chen Q., Fu F. (2019). Multicolor aptasensor based on DNA-induced Au-Ag nanorods for simultaneous and visual detection of inorganic and organic mercury. ACS Omega.

[37] Dong Z., Su R., Fu Y., Wang Y., Chang L. (2024). Recent progress in DNA biosensors for detecting biomarkers in living cells. ACS Biomater. Sci. Eng..

[38] Park G., Park H., Park S.C., Jang M., Yoon J., Ahn J.H., Lee T. (2023). Recent developments in DNA-nanotechnology-powered biosensors for Zika/dengue virus molecular diagnostics. Nanomaterials.

[39] Singh R.R., Patel M.K. (2026). Zinc-doped MgO nanohybrids enable sensitive Salmonella typhimurium biosensing. Talanta.

[40] He X., Han H., Shi W., Dong J., Lu X., Yang W., Lu X. (2020). A label-free electrochemical DNA biosensor for kanamycin detection based on diblock DNA with poly-cytosine as a high affinity anchor on graphene oxide. Anal. Methods.

[41] Miyake Y., Togashi H., Tashiro M., Yamaguchi H., Oda S., Kudo M., Tanaka Y., Kondo Y., Sawa R., Fujimoto T. (2006). MercuryII-mediated formation of thymine-Hg^II^-thymine base pairs in DNA duplexes. J. Am. Chem. Soc..

[42] Deng L., Ouyang X., Jin J., Ma C., Jiang Y., Zheng J., Li J., Li Y., Tan W., Yang R. (2013). Exploiting the higher specificity of silver amalgamation: Selective detection of mercury(II) by forming Ag/Hg amalgam. Anal. Chem..

[43] Kamal A., She Z., Sharma R., Kraatz H.B. (2017). Interactions of Hg(ii) with oligonucleotides having thymine-thymine mispairs. Optimization of an impedimetric Hg(ii) sensor. Analyst.

[44] Huang L., Li P., Lin C., Wu Y., Chen Z., Fu F. (2021). DNA-templated fluorescent silver nanoclusters on-off switch for specific and sensitive determination of organic mercury in seafood. Biosens. Bioelectron..

[45] Dragomir I., Schiopu I., Asandei A. (2025). Probing the stability of Hg^2+^-mediated self-duplexes: Effects of thymine residues position and number via a single-molecule protein nanopore sensing technique. Biosens. Bioelectron..

[46] Liu C.W., Tsai T.C., Osawa M., Chang H.C., Yang R.J. (2018). Aptamer-based sensor for quantitative detection of mercury (II) ions by attenuated total reflection surface enhanced infrared absorption spectroscopy. Anal. Chim. Acta.

[47] Soutenain A., Chorel M., Lavastre E., Macnally S., Mireles M., Rigatti A., Ducros C., Dublanche-Tixier C. (2024). Broadband optical spectral shift analysis of dielectric coatings. Opt. Lett..

[48] Kosame S., Josline M.J., Lee J.H., Ju H. (2024). Anomalous spectral shift of localized surface plasmon resonance. Nanoscale Adv..

[49] Gu X., Ning X., Fu J., Geng S., Zhao Q., Cai D., Zeng Y., Xu Y., Tian S. (2026). SERS-based mercury ion “on-off” dual-mode sensor using a silver cavity array substrate. Talanta.

[50] Wang N., Cao P., Ma H., Lin M. (2021). How Stabilizers and Reducing Agents Affect the Formation of Nanogold Amalgams. Langmuir.

[51] Yang J.Y., Jia X.D., Wang X.Y., Chen M.L., Yang T., Wang J.H. (2020). Mercury speciation based on mercury-stimulated peroxidase mimetic activity of gold nanoparticles. Analyst.

[52] Salah D., Moghanm F.S., Arshad M., Alanazi A.A., Latif S., El-Gammal M.I., Shimaa E.M., Elsayed S. (2021). Polymer-peptide modified gold nanorods to improve cell conjugation and cell labelling for stem cells photoacoustic imaging. Diagnostics.

[53] Wang W., Ding X., Xu Q., Wang J., Wang L., Lou X. (2016). Zeta-potential data reliability of gold nanoparticle biomolecular conjugates and its application in sensitive quantification of surface absorbed protein. Colloids Surf. B Biointerfaces.

[54] Cai B., Ren T., Yu X., Lv W., Liang Y. (2024). Aptamer-functionalized gold nanoparticles for mercury ion detection in a colorimetric assay based on color change time as signal readout. Microchim. Acta.

[55] Ulloa-Gomez A.M., Lucas A., Koneru A., Barui A., Stanciu L. (2023). Simultaneous colorimetric and electrochemical detection of trace mercury (Hg^2+^) using a portable and miniaturized aptasensor. Biosens. Bioelectron..

[56] (2025). National Standard of the People’s Republic of China: GB 2762-2025 National Food Safety Standard Maximum Levels of Contaminants in Foods.

[57] (2022). National Standard of the People’s Republic of China: GB 5749-2022 Standards for Drinking Water Quality.

[58] Qi M., Zhang Z., Li L., Mu X., Wang Y. (2023). A sensitive ratiometric fluorescent chemosensor for visual and wearable mercury (II) recognition in river prawn and water samples. Food Chem..

[59] Kim J., Jeon J., Wang C., Chang G.T., Park J. (2022). Asymmetric nanochannel network-based bipolar ionic diode for enhanced heavy metal ion detection. ACS Nano.

[60] Pourreza N., Ghanemi K. (2009). Determination of mercury in water and fish samples by cold vapor atomic absorption spectrometry after solid phase extraction on agar modified with 2-mercaptobenzimidazole. J. Hazard. Mater..

[61] Wang X., Tang P., Liu H., Li Y., Guo H., Li P., Han D., Liu N., Liu L., He B. (2025). Detection of mercury in single mammalian cells at attogram level by SC-ICP-MS. Anal. Chim. Acta.

[62] Liu X., Zhang S., Tan P., Zhou J., Huang Y., Nie Z., Yao S. (2013). A plasmonic blood glucose monitor based on enzymatic etching of gold nanorods. Chem. Commun..

[63] Chen Y., Cheng X., Mo F., Huang L., Wu Z., Wu Y., Xu L., Fu F. (2016). Ultra-sensitive speciation analysis of mercury by CE-ICP-MS together with field-amplified sample stacking injection and dispersive solid-phase extraction. Electrophoresis.

[64] Zhao Y., Zheng J., Fang L., Lin Q., Wu Y., Xue Z., Fu F. (2012). Speciation analysis of mercury in natural water and fish samples by using capillary electrophoresis–inductively coupled plasma mass spectrometry. Talanta.

