# Supplementary material for: Developing an Aptamer-Based Colorimetric Method for Highly Specific Discrimination of Hg2+ and MeHg
Source: Molecules. 2026 Nov 1;31(21):3925. doi: 10.3390/molecules31213925 (PMC13765184; doi:10.3390/molecules31213925)
Supplement: Supplementary file 1 [file molecules-31-03925-s001.zip › molecules-4628918-supplementary.pdf]

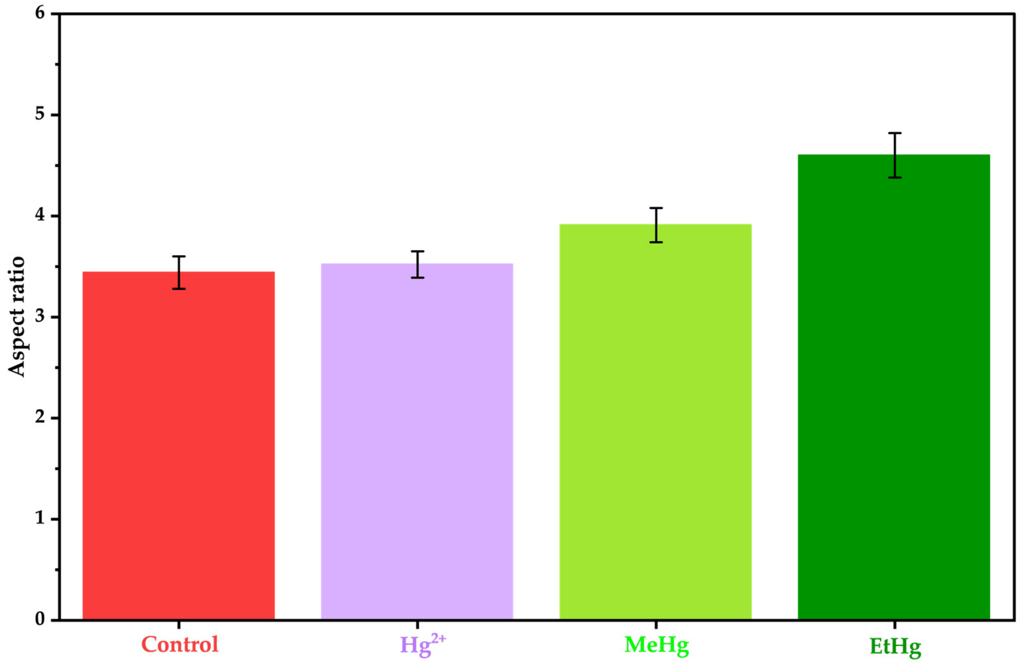


**Figure S1.** The average aspect ratio (AR) of PDDAC-coated AuNRs with/without mercury species.

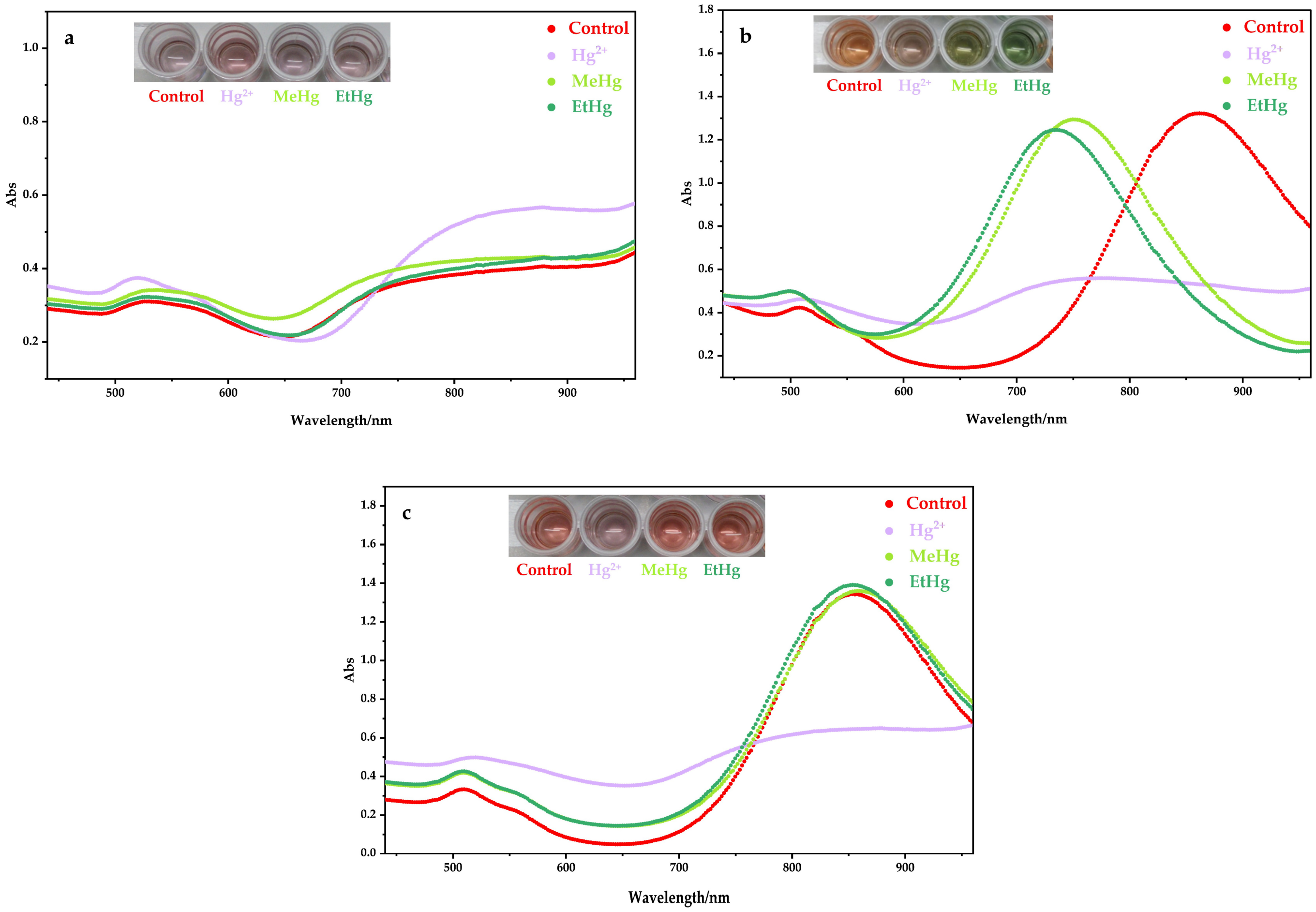


**Figure S2.** Optimization of the reducing agent. (a) TC; (b) $NaBH_4$; (c) AA.

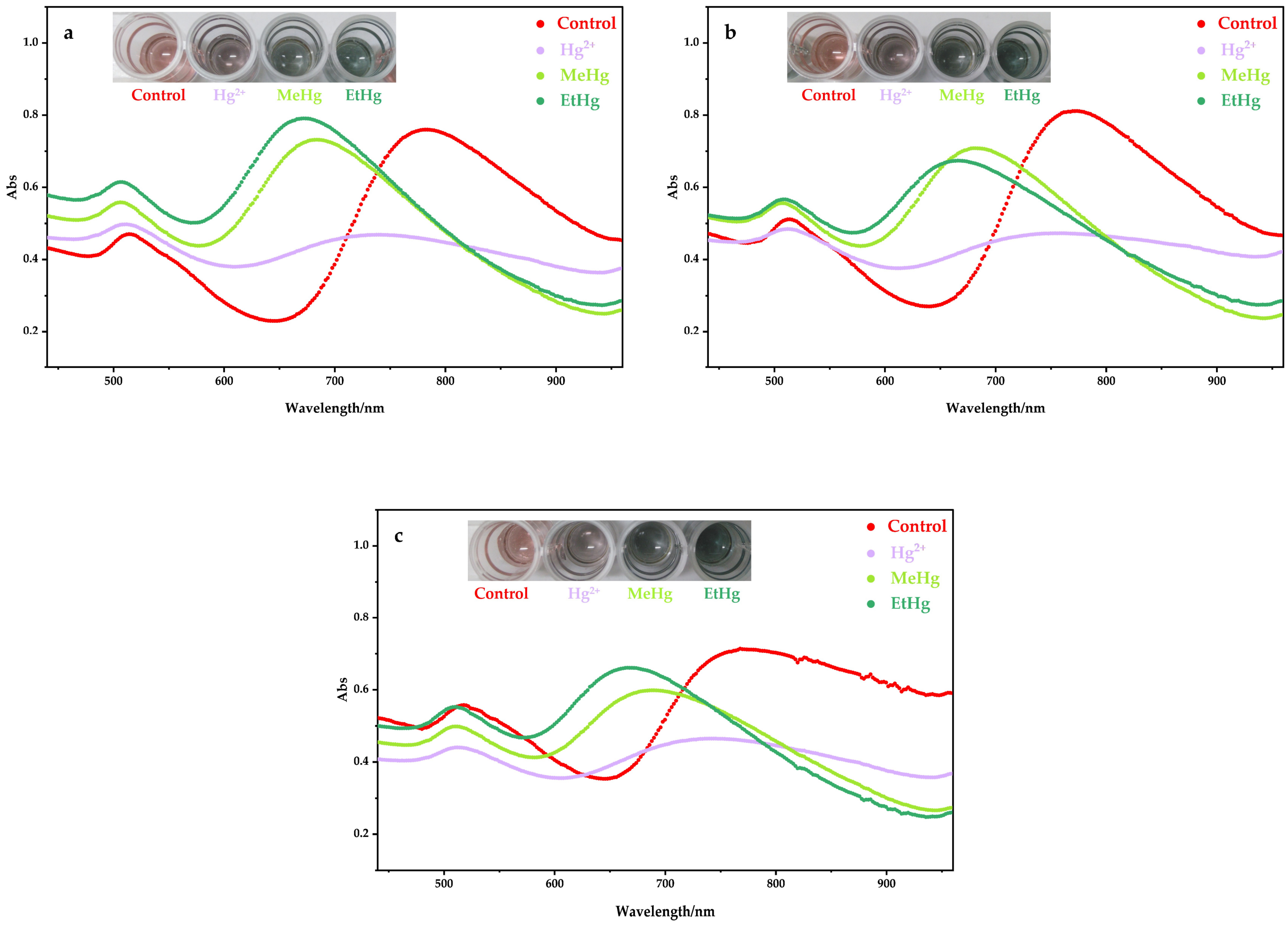


**Figure S3.** Optimization of the NaBH4 concentration. (a) 70 mM; (b) 85 mM; (c) 100 mM.

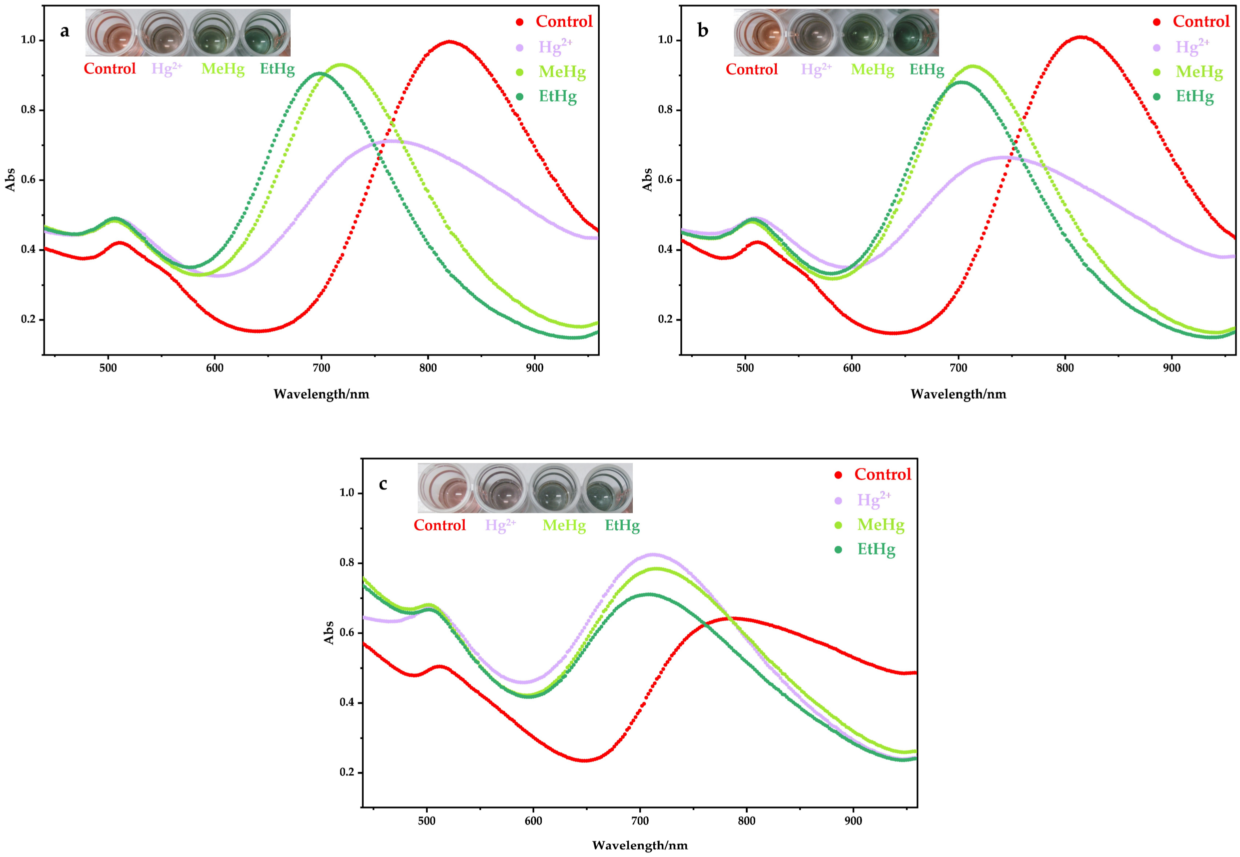

**Figure S4.** Optimization of the pH in the system solution. (a) pH 7.5; (b) pH 8.5; (c) pH 9.5.

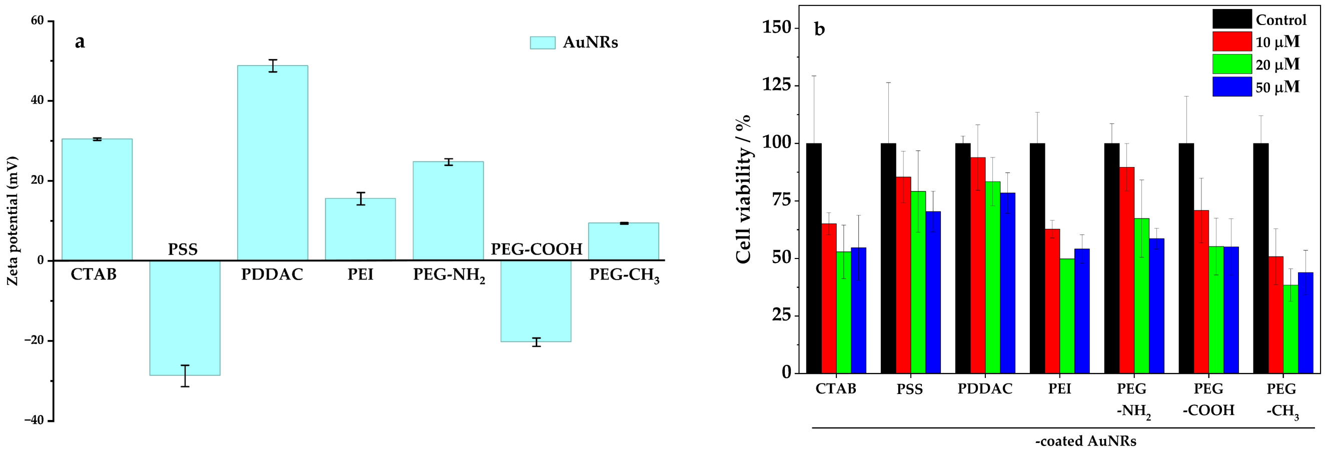


**Figure S5.** The zeta potential and cell viability of AuNRs with seven different surface modifications.

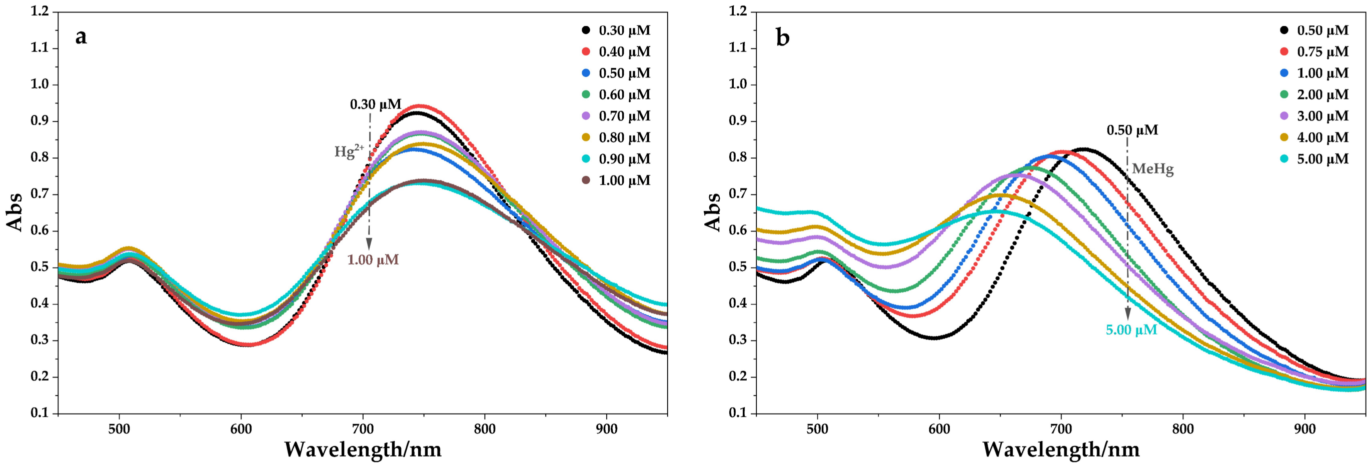


**Figure S6.** The UV-vis absorption spectra of the HT9 DNA aptamer-AuNRs composites in the presence of different concentrations of $Hg^{2+}$ or MeHg.

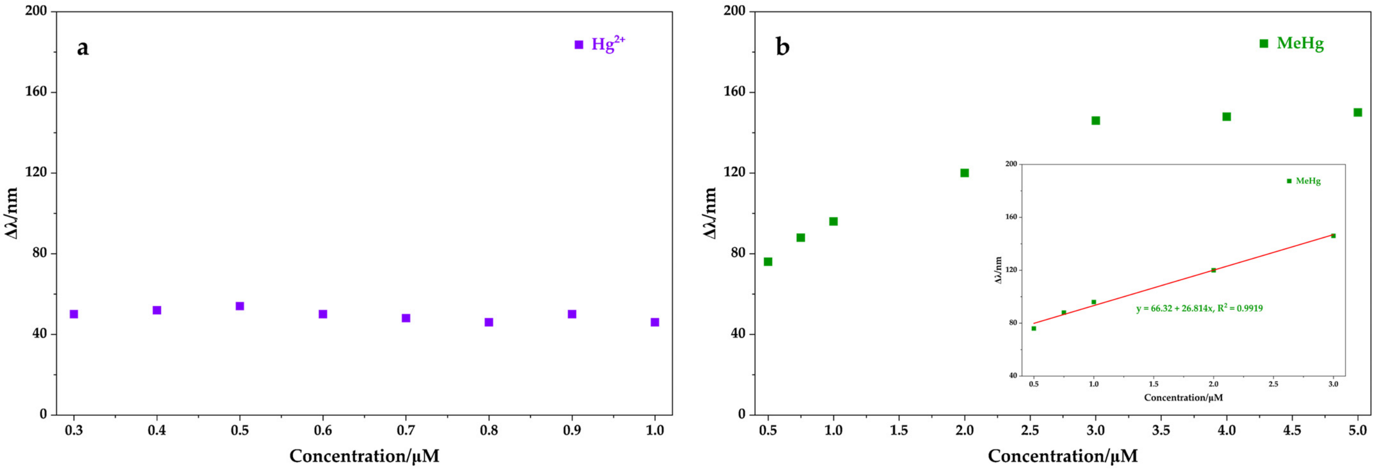


**Figure S7:** The relationship between the peak shift (Δλ) versus concentration in the presence of different concentrations of $Hg^{2+}$ or MeHg.
